# Imaging characteristics of intravascular spherical contrast agents for grating-based x-ray dark-field imaging – effects of concentrations, spherical sizes and applied voltage

**DOI:** 10.1038/s41598-020-66395-x

**Published:** 2020-06-10

**Authors:** Susan Notohamiprodjo, Karla Maria Treitl, Christian Hauke, Sven-Martin Sutter, Sigrid Auweter, Franz Pfeiffer, Maximilian Ferdinand Reiser, Katharina Hellbach

**Affiliations:** 1 Department of Nuclear Medicine, Klinikum rechts der Isar, Technical University of Munich, Munich, Germany; 2 Department of Radiology, University Hospital, LMU Munich, Munich, Germany; 3 Siemens Healthcare GmbH, Forchheim, Germany; 4 Erlangen Centre for Astroparticle Physics, Friedrich-Alexander-University Erlangen-Nuremberg, Erlangen, Germany; 5 Smart Reporting GmbH, Munich, Germany; 6 Department of Physics and Institute of Medical Engineering, Technical University of Munich, Munich, Germany; 7 Department of Diagnostic and Interventional Radiology, University Hospital of Heidelberg, University of Heidelberg, Heidelberg, Germany

**Keywords:** Drug development, Preclinical research, X-rays, Imaging techniques

## Abstract

This study investigates the x-ray scattering characteristics of microsphere particles in x-ray-grating-based interferometric imaging at different concentrations, bubble sizes and tube voltages (kV). Attenuation (ATI), dark-field (DFI) and phase-contrast (PCI) images were acquired. Signal-to-noise (SNR) and contrast-to-noise ratios with water (CNR_w_) and air as reference (CNR_a_) were determined. In all modalities, a linear relationship between SNR and microbubbles concentration, respectively, microsphere size was found. A significant gain of SNR was found when varying kV. SNR was significantly higher in DFI and PCI than ATI. The highest gain of SNR was shown at 60 kV for all media in ATI and DFI, at 80 kV for PCI. SNR for all media was significantly higher compared to air and was slightly lower compared to water. A linear relationship was found between CNR_a_, CNR_w_, concentration and size. With increasing concentration and decreasing size, CNR_a_ and CNR_w_ increased in DFI, but decreased in PCI. Best CNR_a_ and CNR_w_ was found at specific combination of kV and concentration/size. Highest average CNR_a_ and CNR_w_ was found for microspheres in ATI and PCI, for microbubbles in DFI. Microspheres are a promising contrast-media for grating-based-interferometry, if kV, microsphere size and concentration are appropriately combined.

## Introduction

Contrast differences in conventional X-ray imaging rely on the varying degree of attenuation of x-rays penetrating different types of tissue. Contrast is highest in imaging materials with large differences in atomic number, material density, or both, such as bone or lung. However, in clinical radiography structures with only slight density differences, such as the tissue composition within inner organs, need to be examined as well, limiting the usefulness of x-ray attenuation imaging (ATI).

Current research focuses on the phase change when the x-ray beam passes through the imaging object. Besides attenuation, alterations in phase change in different types of soft tissue provide additional contrast information. Here, image contrast is generated from interference of the x-ray wave front, caused by a phase shift of the x-ray wave while passing through the object. The phase of a wave front cannot be measured directly. Therefore, the imaging method requires a translation from phase shift to intensity differences. There are various approaches to retrieve phase information from x-ray imaging, including free-space propagation techniques^1,2^, analyser-crystal-based methods^3,4^, edge illumination^5^ and interferometric methods^6,7^. Among these, grating-based x-ray imaging using Talbot-Lau interferometry, which is applicable to polychromatic laboratory x-ray sources common in clinical settings, has shown promising results in biomedical imaging applications^8–14^. Here, complementary contrast information of three approaches are achieved: the attenuation image (ATI), the phase-contrast image (PCI) and the dark-field image (DFI). Contrast in DFI refers to the extinction of the interference fringes due to small-angle scattering^8,9,15^.

To increase contrast within different types of soft-tissue, applying contrast media with high atomic numbers is common practice^16^. For absorption-based x-ray imaging, iodine-based contrast agents are commonly used to enhance contrast. Intravascular administration of relatively high concentrations and volumes in a short period of time is necessary to create sufficient contrast^17^. Although the safety of x-ray contrast agents has improved significantly during the last decades, these products can cause various adverse reactions^18,19^.

Contrast media, particularly gas-filled microbubble-based agents, are also used to improve ultrasound imaging in order to provide a more accurate diagnosis^20,21^. Microbubbles generate contrast in ultrasound by providing many small compressible regions. They oscillate and amplify the signal reflected back towards the ultrasound transducer. In comparison to conventional iodine based contrast agents, microbubbles are considered to be safer^22–24^. They offer possibilities to develop novel imaging approaches, as they generate contrast through an alternative physical mechanism, creating a signal that can be differentiated from absorption. Microbubbles are spherical regions of differing electron density and function as a cloud of x-ray lenses in x-ray phase-contrast imaging. Thus, refraction in multiple microbubbles scatters x-rays in diverse directions, generating an area of increased signal greater than those of the surrounding tissues^22,25^.

Recently, the use of microbubbles in imaging techniques other than ultrasound have been reported^26^, such as synchrotron-based methods^22^ as well as approaches based on Talbot-Lau interferometry^27^.

However, DFI and PCI signals depend on parameters such as concentration of microbubbles, their sizes and the energy used. Different concentrations of microbubbles and other media with larger spheres than microbubbles have not been investigated in DFI and PCI with different tube voltages yet.

In this explorative study we report x-ray scattering characteristics in direct comparison to various clinically available microbubbles and microspheres for intravascular application in different concentrations and sizes and at different tube voltages for DFI and PCI.

## Results

### Visual analysis

**Figure 1:**
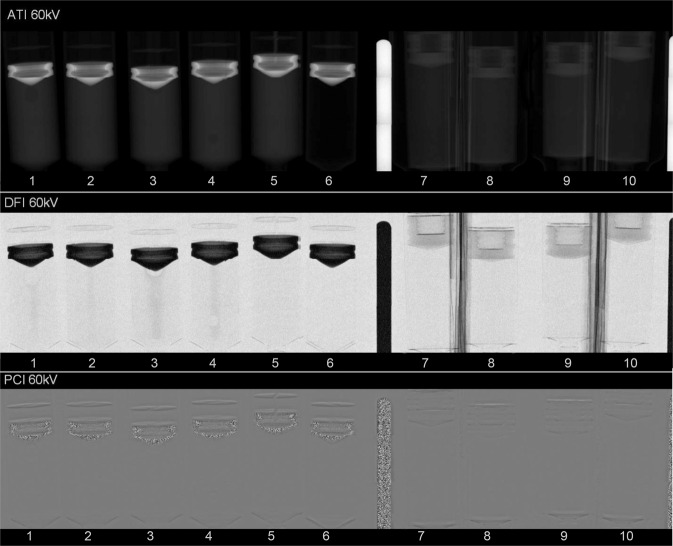
Exemplary image of ATI, DFI and PCI acquired at a tube voltage of 60 kV. From left to right on position 1–4: Microbubbles with concentrations of 0.64, 0.32, 3.2 and 1.6 µg/ml. On position 5–6: H_2_O and air in empty vial as reference material. On position 7–10: Microspheres with 1100, 900, 700, 500 µm size. The material between position 6–7 made of lead, respectively, between positions 7–8 and 9–10 made of paperboard, is to stabilize the syringes during the examination. Reconstruction and postprocessing of acquired data were performed using a combination of C++ and Matlab R2016A (Mathworks Inc, MA) software packages (see “Scanner setup and image acquisition” section in Materials & Methods).

In ATI, all microbubble and microsphere samples show a notably greater signal in comparison with air at all tube voltages. In DFI a distinct enhanced signal in all media samples at all preset tube voltages compared to reference air was observed. In PCI, edge enhancement of the sample vials and enhanced background signal outside the vials is markedly visible. Compared to water, the DFI signals of all media samples were apparently lower (Fig. 1).

### Reference water and air

**Figure 2:**
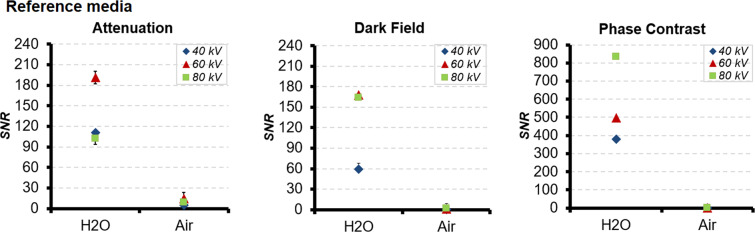
SNR of the reference materials water and air at tube voltages 40 kV, 60 kV and 80 kV in ATI, DFI and PCI.

The SNR in ATI, DFI, PCI of reference water and of air at 40 kV, 60 kV and 80 kV is summarized in Table 1 and is displayed in a diagram for each imaging modality ATI, DFI and PCI (Fig. 2).

**Table 1:** SNR of the reference water and air at 40 kV, 60 kV and 80 kV applied voltage in ATI, DFI and PCI.

		SNR		
	Voltage	ATI	DFI	PCI
Water	40 kV	110.82 ± 3.25	59.34 ± 9,02	378.72 ± 1.29
	60 kV	191.24 ± 8.86	167.89 ± 2.68	497.12 ± 1.87
	80 kV	101.91 ± 8.17	164.12 ± 2.34	833.28 ± 0.79
Air	40 kV	4.78 ± 1.90	0.25 ± 5.80	0.60 ± 1.55
	60 kV	14.37 ± 3.02	1.18 ± 0.42	0.92 ± 2.92
	80 kV	8.58 ± 1.66	1.80 ± 1.16	1.23 ± 1.62

### Microbubbles

**Figure 3:**
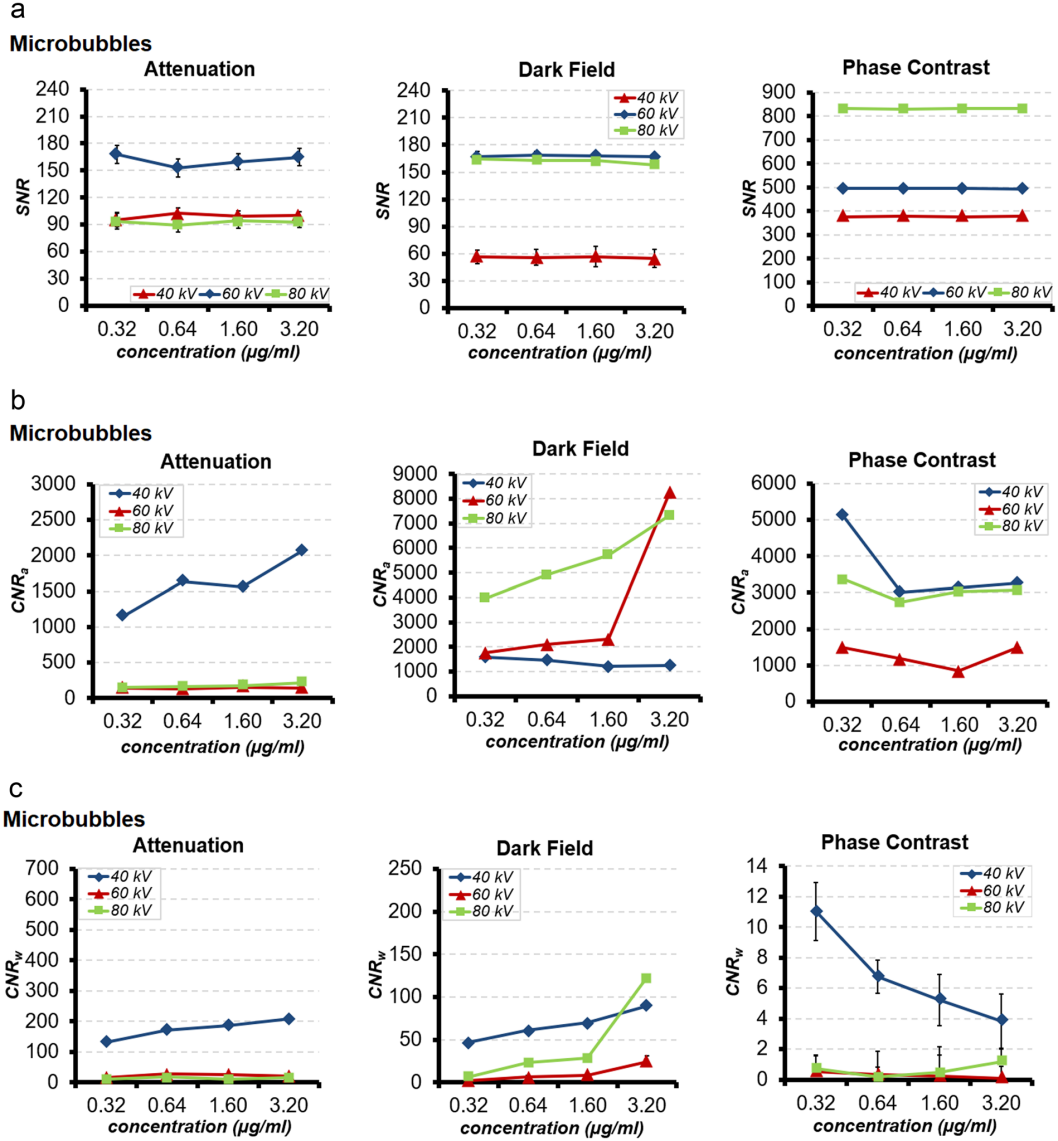
(**a**) SNR (**b**) CNR_a_ and (**c**) CNR_w_ of microbubbles with concentrations of 0.32, 0.64, 1.6 and 3.2 µg/ml at tube voltages of 40, 60 and 80 kV in ATI, DFI and PCI.

In ATI, DFI and PCI linear relationships between SNR and 0.32, 0.64, 1.6 and 3.2 µg/ml microbubbles concentrations at 40, 60 and 80 kV applied voltages were observed (Fig. 3a).

In ATI, a significant decrease of SNR was found only when increasing microbubbles concentration from 0.32 to 0.64 µg/ml at 60 kV applied voltage (p = 0.02). However, changes of SNR at other microbubble concentrations and other applied voltages were not significant (p = 0.26 at 40 kV; p = 0.29 at 60 kV; p = 0.57 at 80 kV). In general, the SNR of microbubbles was independent from the concentrations used in this study at all applied voltages (Fig. 3a). However, SNR is significantly dependent on applied voltages throughout all microbubble concentrations, with a substantial increase from 40 to 60 kV and substantial decrease from 60 to 80 kV as well as from 40 to 80 kV (p < 0.001) (Fig. 3a). Thus, the SNR of microbubbles in ATI highly depends on the applied voltage rather than microbubbles concentration. The most effective combination of applied voltage and microbubbles concentration was found at 60 kV and 0.32 µg/ml.

Similar to ATI, in DFI and PCI no significant changes of SNR was detected with increasing microbubble concentration at all applied voltages (p = 0.458 for DFI, p = 0.981 for PCI) (Fig. 3a). However, SNR in microbubbles significantly changed in varying applied voltages (p < 0.001) (Fig. 3a). In DFI, SNR substantially increased from 40 to 60 kV, 40 to 80 kV and significantly decreased from 60 to 80 kV. In PCI SNR substantially increased from 40 to 60 kV, 40 to 80 kV and 60 to 80 kV.

Comparison of SNR between air as reference and microbubbles, each with concentrations of 0.32, 0.64, 1.6 or 3.2 µg/ml and at 40, 60 and 80 kV applied voltages in ATI, DFI and PCI revealed significant higher SNR in microbubbles than in air (p < 0.001). The highest SNR was found in PCI, followed by DFI and ATI. In ATI comparison of SNR between water as reference and microbubbles showed slightly lower SNR in microbubbles than in water at all concentrations and applied voltages, but the differences of SNR were all statistically significant (p < 0.001). In DFI and PCI the comparison of SNR between water as reference and microbubbles showed similar SNR at all concentrations and applied voltages (p = 0.22–0.99).

MNOVA analysis of CNR_a_ of microbubbles at all concentrations and all applied voltages in ATI, DFI and PCI showed significant interdependence between concentration and applied voltage (p < 0.001). An increase in microbubble concentration resulted in significant enhancement of CNR_a_ in ATI at 40 kV (p < 0.001) (Fig. 3b). A linear relationship of CNR_a_ for microbubbles in ATI at 60 and 80 kV was also detected. However, the changes of CNR_a_ at concentrations of 0.32, 0.64, 1.6 and 3.2 µg/ml were minimal, but still statistically significant (p = 0.04 – <0.001). The main contributor to enhancement of CNR_a_ in ATI was applied voltage (p < 0.001). The most effective combination of applied voltage and microbubble concentration was found at 40 kV and 3.2 µg/ml (Fig. 3b). In DFI and PCI, the CNR_a_ of microbubbles showed a higher degree of concentration dependence than in ATI with significant changes of CNR_a_ with increasing microbubble concentration at all applied voltages (p < 0.001) (Fig. 3b). The most effective combination of applied voltage and microbubble concentration was found at 60 kV and 3.2 µg/ml in DFI. In PCI, the highest CNR_a_ of microbubbles was found at 40 kV and 0.32 µg/ml (Fig. 3b).

MNOVA analysis of CNR_w_ of microbubbles at all concentrations and applied voltages in ATI, DFI and PCI showed significant interdependence between concentration and applied voltage (p < 0.001). An increase in microbubble concentration resulted in significant enhancement of CNR_w_ in ATI at 40 kV (p < 0.001) (Fig. 3c). A linear relationship between CNR_w_ and microbubble concentration in ATI at 60 and 80 kV was also detected. However, changes in CNR_w_ among all concentrations were minimal, but still statistically significant (p = 0.04 – <0.001). The main contributor to enhancement of CNR_w_ in ATI was applied voltage (p < 0.001). The most effective combination of applied voltage and microbubble concentration was found at 40 kV and 3.2 µg/ml (Fig. 3c). In DFI, the CNR_w_ of microbubbles was dependent on concentration to a higher degree than in ATI, with a significant enhancement of CNR_w_ with increasing microbubble concentrations at all applied voltages (p < 0.001) (Fig. 3c). The most effective combination of applied voltage and concentration of microbubbles was measured at 80 kV and 3.2 µg/ml. In PCI, a linear relationship between CNR_w_ and microbubble concentration at all applied voltages was found. However, in contrast to ATI and DFI an increase in microbubble concentration resulted in a significant decrease of CNR_w_ in PCI at 40 and 60 kV (p < 0.001) (Fig. 3c). At 80 kV, a slightly significant enhancement of CNR_w_ with increasing microbubble concentration was registered (p = 0.01) (Fig. 3c). The most effective combination of applied voltage and microbubble concentration was measured at 40 kV and 0.32 µg/ml.

### Microspheres

**Figure 4:**
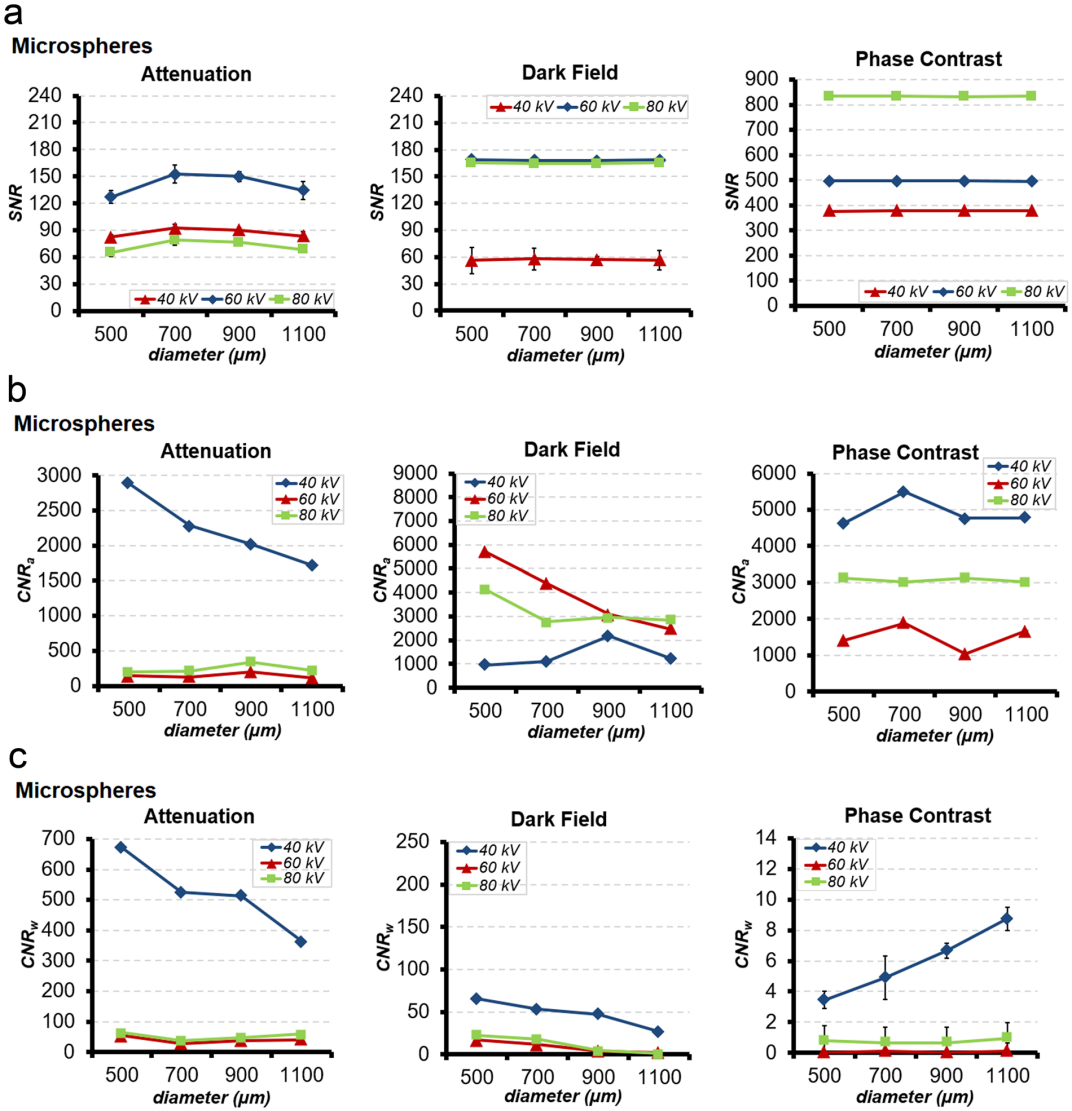
(**a**) SNR (**b**) CNR_a_ and (**c**) CNR_w_ of microspheres with sizes of 1100, 900, 700 and 500 µm at tube voltages of 40, 60 and 80 kV in ATI, DFI and PCI.

In ATI, DFI, and PCI, linear relationships between SNR and microsphere size 500, 700, 900 and 1100 µm at 40, 60 and 80 kV were registered with an almost flat course of the signal intensity (Fig. 4a).

In ATI, a significant dependence of SNR on all microsphere sizes and applied voltages was observed (p < 0.001) (Fig. 4a). The highest SNR was registered at a microsphere size of 700 µm at all applied voltages. The changes of SNR with increasing microsphere size at all applied voltages were significant (p < 0.001). The variation in applied voltage had a greater effect on SNR than the microsphere size (Fig. 4a). The most effective combination of applied voltage and microsphere size in ATI was found at 60 kV and 700 µm. By contrast, in DFI and PCI, no significant dependence of SNR on microsphere size at all applied voltages was observed (p = 0.98 for DFI, p = 0.99 for PCI). However, a significant dependence of SNR on the applied voltages at all microsphere sizes was evident (p < 0.001) (Fig. 4a). In DFI, the highest SNR was registered at 60 kV, followed by 80 kV. A noticeable lower SNR was registered at 40 kV. In PCI, the highest SNR was registered at 80 kV, followed by 60 and 40 kV. In DFI and PCI significant changes in SNR were measured in response to changes in applied voltage from 40 to 60, 60 to 80 and 40 to 80 kV (p < 0.001).

Similar to microbubbles, in ATI, DFI, and PCI, the comparison of SNR between reference air and microspheres, each with microsphere sizes of 500, 700, 900 or 1100 µm, showed significant higher SNR from microspheres than air at all applied voltages (p < 0.001). The highest SNR was found in PCI, followed by DFI, then ATI. The average SNR of microspheres in DFI was slightly higher than that in ATI, but still statistically significant (p < 0.001).

Comparison of SNR between reference water and microspheres showed slightly lower SNR of microspheres than water for all microsphere sizes and all applied voltages. The differences in SNR were all statistically significant (p < 0.001). In DFI and PCI, the SNR of water and microsphere at all sizes were comparable at all applied voltages (p = 0.32–0.99).

MNOVA analysis of CNR_a_ from microspheres showed significant interdependence between the microsphere size and applied voltage (p < 0.001) (Fig. 4b). A linear relationship between CNR_a_ and microsphere sizes at 60 and 80 kV was identified. However, the changes of CNR_a_ among all microsphere sizes were minimal, but still statistically significant (p = 0.01 – <0.001). The main contributor to increasing CNR_a_ in ATI was applied voltage (p < 0.001). The most effective combination of applied voltage and microsphere size was measured at 40 kV and 500 µm (Fig. 4b). In DFI and PCI, CNR_a_ showed higher dependency on microsphere size than in ATI at all applied voltages (p < 0.001) (Fig. 4b). The most effective combination of the applied voltage and microsphere size was registered at 60 kV and 500 µm (Fig. 4b). In PCI, highest CNR_a_ of microspheres was measured at 40 kV and 700 µm size (Fig. 4b).

MNOVA analysis of CNR_w_ showed significant interdependence between the microsphere size and applied voltage (p < 0.001). An increase in microsphere size resulted in a significant decrease of CNR_w_ in ATI at 40 kV applied voltage (p < 0.001) (Fig. 4c). A linear relationship between CNR_w_ and microsphere size in ATI at 60 and 80 kV was detected. However, the changes in CNR_w_ among all microsphere sizes were minimal, but still statistically significant (p < 0.001). The main contributor to enhancement of CNR_w_ in ATI was applied voltage (p < 0.001). The most effective combination of applied voltage and microsphere size was found at 40 kV and 500 µm (Fig. 4c). The variation of the applied voltage, 40 to 60 kV, 40 to 80 kV induced a pronounced reduction in CNR_w_ signal. In DFI, CNR_w_ showed higher dependency on microsphere size than in ATI. With increasing microsphere size CNR_w_ decreased significantly at all applied voltages (p < 0.001) (Fig. 4c). The most effective combination of applied voltage and microsphere size was found at 40 kV and 500 µm. In PCI, a linear relationship of CNR_w_ with microsphere size at all applied voltages was also measured. In contrast to ATI and DFI, CNR_w_ in PCI at 40 kV increased significantly with increasing microsphere size (p < 0.001) (Fig. 4c). However, the increase of CNR_w_ with increasing microsphere size was not significant at 60 and 80 kV (p = 0.60) (Fig. 4c). By contrast, CNR_w_ at 60 kV and at 80 kV among all microsphere sizes was significantly different (p < 0.001). At 80 kV CNR_w_ was higher than at 60 kV (Fig. 4c). The most effective combination of applied voltage and microsphere size was measured at 40 kV and 1100 µm.

### Comparison between the investigated contrast media

**Figure 5:**
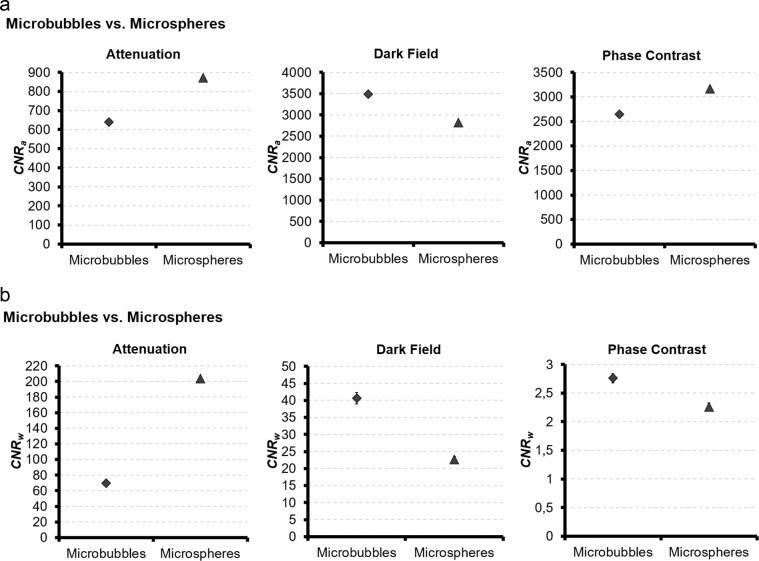
Comparison between all contrast media. (**a**) The average CNR_a_ of microbubbles and microspheres in ATI, DFI and PCI. (**b**) The average CNR_w_ of microbubbles and microspheres in ATI, DFI and PCI. The average CNR_a_ value was obtained by averaging the CNR_a_ values of all microbubble concentrations at all voltages for each imaging technique ATI, DFI and PCI, respectively for microspheres, by averaging the CNR_a_ values of all microsphere sizes at all voltages for each imaging technique ATI, DFI and PCI. The same procedure was performed for CNR_w_.

In ATI, DFI and PCI the MNOVA multi factor analysis of the investigated contrast media showed a significant interdependence between the investigated contrast media, their concentrations or microsphere sizes and applied voltages (p < 0.001). Comparison of the average CNR_a_ in ATI revealed that microspheres had the highest CNR_a_. In DFI microbubbles showed the highest CNR_a_. In PCI microspheres showed the highest CNR_a_ (Fig. 5a).

Comparison of the average CNR_w_ in ATI revealed that microspheres had the highest CNR_w_, followed by microbubbles. In DFI microbubbles showed the highest CNR_w_. In PCI microbubbles showed the highest CNR_w_ (Fig. 5b).

## Discussion

The aims of the study are (1) to examine the imaging characteristics and signal behaviour of the investigated and for intravascular injection clinically approved spherical media with variation of applied voltages, changes in microbubble concentrations and variations in the size of microspheres, (2) to examine the interdependence of the factor concentration, diameter of the spherical media and the applied voltages, and (3) to extract the most effective combination of those factors to generate a signal potentially useful for a clinical setting.

For all sample media investigated in this study, SNR, which is associated with visibility, shows the highest value at a tube voltage of 60 kV in ATI, and at a tube voltage of 80 kV in DFI as well as PCI. At 40 kV SNR was significantly lower. SNR was similar in DFI at tube voltages of 60 kV and 80 kV. Thus, concerning SNR, 40 kV tube voltage does not seem to be optimal for the experimental setting. SNR of all sample media was significantly higher than air, but slightly lower than water at all preset tube voltages in ATI, DFI and PCI. Similar results were reported by Velroyen *et al*. in studies with fixated or jellified microbubbles and jellified water^27^. In PCI, the background signal outside the vials was high, even though there was no scattering media (Fig. 1). A similarly high background signal was also seen in the figures reported previously^14,27–29^. Differential-phase images are depicted in this work without any integration of the differential phase. In general, background of PCI is only zero in absence of any objects or in absence of any change of electron density and exact phase-stepping sampling. Since an exact phase-stepping sampling cannot be achieved for a pre-clinical prototype with inevitable external influences (e.g. vibrations) and system component inaccuracies, there will be a background offset in PCI. The dependency of phase-step position deviations and signal offsets in PCI is described in detail by Hauke *et al*.^30^. The application of image subtraction with and without sample may be helpful.

Microbubbles and microspheres show robust CNR_a_ for DFI and PCI compared to ATI at all preset tube voltages and for all investigated concentrations and sizes. In contrast, microbubbles and microspheres demonstrate a significant loss of CNR_w_ in DFI and PCI compared to ATI, indicating a negative contrast. At optimal tube voltages of 60 kV and 80 kV, CNR_a_ and CNR_w_ of microbubbles and microspheres in DFI increase continuously with increasing concentration and decreasing bubble sizes. This indicates a significant augmentation of the reflectivity of scattered x-rays and demonstrates that microbubble concentration correlates to CNR_a_ and CNR_w_ in DFI. This finding is also supported by the results reported by Millard *et al*. by using the Monte Carlo model simulation^29,31^ and reported by Velroyen *et al*.^27^. On the other hand, the CNR_a_ and CNR_w_ decrease with increasing microsphere sizes in DFI at optimal tube voltage of 60 kV and 80 kV. This is probably due to the reduction of the reflectivity of scattered x-rays through the large microsphere surface boundary^32,33^. The dark-field contrast sensitivity for different microstructure particles is dependent on the imaging-system parameters^32^ and thus varies for different bubble sizes in one system^32,33^. A simulation model (displayed in a diagram by Velroyen *et al*.^27^) describes the dependency of dark-field signal on the diameter of the microbubbles. According to this model, the DFI signal values of large sized microspheres and of microbubbles measured in this study would be at the plateau and ascending part of the simulation curve, respectively. The CNR_a_ and CNR_w_ of both, microbubbles and microspheres, decrease slightly in PCI with increasing concentration and decreasing bubble size, indicating a negative contrast.

An overview of the best combination of microbubble concentrations, microsphere sizes and voltages in ATI, DFI and PCI to achieve best signal in CNR_a_ and CNR_w_ is presented in Table 2.

**Table 2:** Overview of the most effective combination between microbubble concentrations, microsphere sizes and voltages in ATI, DFI and PCI with highest signal in CNR_a_ and CNR_w_.

	Microbubbles			Microspheres		
	Contrast	Voltage	Concentration	Contrast	Voltage	Size
ATI	CNR_a_	40 kV	3.2 µg/ml	CNR_a_	40 kV	500 µm
	CNR_w_	40 kV	3.2 µg/ml	CNR_w_	40 kV	500 µm
DFI	CNR_a_	60 kV	3.2 µg/ml	CNR_a_	60 kV	500 µm
	CNR_w_	80 kV	3.2 µg/ml	CNR_w_	40 kV	500 µm
PCI	CNR_a_	40 kV	0.32 µg/ml	CNR_a_	40 kV	700 µm
	CNR_w_	40 kV	0.32 µg/ml	CNR_w_	40 kV	1100 µm

Many preclinical investigations^11,28,34–38^ demonstrate a huge potential of x-ray grating-based interferometric phase-contrast and dark-field imaging by improving the soft-tissue contrast as compared to attenuation-based imaging. For further improvement of contrast in grating-based interferometric phase-contrast and dark-field imaging, previous studies investigated microbubble-based contrast agents and performed Monte Carlo model simulation studies with microbubbles^22,25,27,31^. They suggest that microbubbles are suitable contrast agent for this imaging method.

Based on the investigation shown in this study, our data suggests that the clinically applicable ultrasound microbubble contrast agent might be a useful contrast agent for grating-based phase-contrast-imaging in a clinical diagnostic setting. Small sizes of microspheres might be a useful agent for clinical interventional procedures with positive contrast enhancement in DFI and with negative contrast enhancement in PCI.

In this study the imaging characteristics of microbubble ultrasound contrast media and microsphere particles, acquired with a preclinical dark-field and phase-contrast imaging scanner, are reported. The imaging setup was successfully used with idealized parameters for preclinical animal investigations published previously^39–41^. This study was challenging, as the bubbles float. To avoid this, Millard *et al*.^25,31^ and Velroyen *et al*.^27^ added a viscous suspension to the microbubble contrast agent solution to fix the bubbles. However, under real clinical conditions the microbubbles are likely to float in the blood stream or in the intercellular space. In this study, we refrained from the use of fixation or jellification of the microbubbles in order to preserve the chemical and osmotic environment as well as for the sake of mimicking real clinical applications. The microspheres used in this study came in 1 ml prefilled products, suspended in a small amount of viscous solution to keep them stable. Therefore, fixation or jellification of microspheres is not necessary. The sample media remained homogeneous during and after the acquisition without recognizable sedimentation of the microspheres at the bottom of the vial (Fig. 1). This indicates that the microbubbles and microspheres maintain their residual kinetic energy supplied during the shaking procedures and were in a thermodynamically equilibrium-like status.

In this study one type of microbubble (SonoVue) and one type of microsphere (Embozene) was used. However, microbubbles sold by other vendors (for example Vevo Micromarker (Visual Sonics) or Optison (GE Healthcare) and microspheres made of other materials such as gelfoam, resin, polyvinylalcohol or gelatine were not available for this study.

The microbubbles and the microspheres used in this study were all with concentrations or sizes usually applied in the clinical routine. Future studies investigating other type of microbubbles, particularly those containing therapeutic agents, finer sampling of microbubble concentrations to determine the best dose, other sizes of microspheres, variation of microsphere density in a unit volume for clinical interventional procedures, examinations of blood vessels in various dimensions, particularly in flowing blood, are needed.

Altogether, this study is highly experimental, but encouraging for further optimization of *in-vivo* visibility, e.g. faster acquisition schemes for *in vivo* dynamic imaging, an interferometer that is more sensitive to smaller refraction angles by using higher Talbot orders.

## Materials & Methods

### Spherical media

Two media with different spherical content, routinely used in clinical ultrasound and interventional radiology, were investigated in this study.

SonoVue (Bracco International B.V., Amsterdam, Netherlands) is a suspension of microbubbles consisting of phospholipid shell filled with stabilized sulfur hexafluoride. After reconstitution of the agent by adding 0.9% saline solution to the powder-like lyophilisate, the concentration was 8 µl powder per ml contrast medium, providing a bubble concentration of up to 5 × 10^8^ per ml^42^. The mean diameter of the microbubbles is 2.5 µm with more than 90% smaller than 8 µm^42^.

Embozene microspheres (CeloNova BioSciences Inc., San Antonio, USA) are spherical, tightly calibrated, biocompatible, non-resorbable, hydrogel microspheres coated with an inorganic perfluorinated polymer in a range of sizes suitable for embolic therapy. They were available as 1 ml prefilled products, suspended in a non-pyrogenic, sterile transport solution of physiological saline. For interventional purposes, these microspheres have to be diluted using the provided 6 ml physiological saline solution and need to be added to iodine-based contrast media before use.

In this study, microbubbles with a concentration of 0.32 µg/ml, 0.64 µg/ml, 1.6 µg/ml, 3.2 µg/ml and microspheres with nominal sizes of 500 µm, 700 µm, 900 µm and 1100 µm were investigated. The microsphere sizes correspond to their diameter. For contrast comparison, reference samples of pure water and air (empty vial) were measured in identical 20 ml plastic vials of 19.13 mm inner diameter in the cylindrical part.

### Scanner setup and image acquisition

The measurements were performed using a prototype preclinical large-object dark-field and phase-contrast scanner with a conventional x-ray tube, a Talbot-Lau interferometer and a flat-panel detector. The prototype is part of a research project cluster and is not commercially available. This machine was successfully applied for animal and human sample investigations published previously^39–41^. In this preclinical study approval by the local ethical review board was waived, because the experimental setting was neither on human nor on animals.

The interferometer comprises of three gratings manufactured by the LIGA process (Karlsruhe Institute of Technology (KIT), Germany): a source grating (G0, gold, height 275 µm, period 11.54 µm), a reference grating (G1, gold, height 6.37 µm, period 3.39 µm, π/2 phase shift at 62.5 keV to the x-ray wave front) and an analyser grating (G2, gold, height 190 µm, period 4.8 µm). The G1 and G2 gratings each consist of eight linearly arranged tiled half-tiles of 5.0 × 2.5 cm^2^. The G0-G1 and G1-G2 distances are 981 mm and 410 mm, respectively. The x-ray source Siemens Gigalix x-ray Tube (Siemens Healthineers GmbH, Erlangen, Germany) has a tungsten anode with a filtration of 0.3 mm of copper and a focal spot size of 0.4 mm (IEC 60336). A Xineos 1515 C flat-panel detector (Teledyne DALSA, Waterloo, Canada) was used with a region of interest of 800 × 800 pixels with a pixel pitch of 99 µm. It represents approximately one quarter of the detector area. The conversion material is caesium iodine. Images were acquired at 200 mA tube current and 40 kV, 60 kV and 80 kV voltage, respectively. The effective energies for the different tube voltages are: 40 kV: 32.6 keV, 60 kV: 43.4 keV and 80 kV: 52.0 keV and were calculated using the spectrum calculator provided by manufacturer. The samples were placed on an acrylic glass plate table positioned directly before the reference grating G1. To acquire absorption, dark-field and phase contrast images, phase-stepping method according to Weitkamp *et al*.^8^ and Pfeiffer *et al*.^9,40^ was applied. Exposure time per phase-step image was 50 ms, time for G2 stepping and detector readout between the frames was 100 ms, yielding a total scan time for one FOV scan of approximately 1.2 s by using 8 phase-stepping of the analyser grating. Reconstruction and postprocessing of acquired data were performed using a combination of C + + and Matlab R2016A (Mathworks Inc, MA) software packages provided by the manufacturer. According to the manufacturer, the implemented reconstruction algorithm was improved by implementing a correction algorithm which minimize the cost functions separately for the attenuation and dark-field images by simultaneously adjusting the phase-step positions of the reference and object scan, and thus minimize the moiré artefacts due to imprecisions during the phase-stepping process^35,36^. The reconstruction runtime was about 2 s per tile on an Intel CoreTM i7-6700HQ processor. Absorption-based, dark-field and differential phase-contrast images were obtained^8,9,15^ in the same session.

### Data analysis

To evaluate visibility, the signal-to-noise ratio (SNR) was calculated using the definition

$$SNR=\frac{{\mu }_{media}}{{\sigma }_{bg}}$$

To obtain a quantitative measurement of the contrast in the microbubble images, the contrast-to-noise ratio (CNR) of the attenuation-based (ATI), dark-field (DFI) and phase-contrast (PCI) images was calculated as

$$CNR=\frac{|{\mu }_{media}-{\mu }_{ref}|}{\sqrt{{\sigma }_{media}^{2}+{\sigma }_{ref}^{2}}}$$

µ_media_ and µ_ref_ denote the average of the ATI, DFI and PCI signal in a certain region of interest (ROI) in the media sample and the reference (ref) sample, respectively. σ_media,_ σ_ref_ and σ_bg_ represent the standard deviation of contrast agent, reference sample, and background region, respectively. The ROIs were manually chosen areas of 64 × 64 pixels in the centre of the cylindrical part of the vials as well as outside the vials for background measurement.

To achieve correct measurement of the content within the vial without the signal contribution of the vial itself, signal subtraction is necessary. The signal intensity of the background measured outside the vials was subtracted from the signal intensity of the empty vial. This calculated signal of the vial itself is subtracted from the signal intensities of the samples of interest.

CNR_a_ denotes the CNR with reference sample air and CNR_w_ the CNR with reference sample water. For clinical application the information of SNR, CNR_a_ and CNR_w_ are equally important. SNR indicates the visibility of the signal. CNR_a_ gives contrast information in organs surrounded mostly by air such as in lung parenchyma or in the digestive tract. CNR_w_ gives contrast information in soft tissues.

Means and standard deviations for signal intensity and contrast were calculated. Results were tested for statistical significance with a significance level (α) of 0.05. Multivariate analysis of variance (MNOVA) was applied to detect significant factors – such as the contrast media, their concentrations, their spherical sizes and applied voltages – which contribute to the signal enhancement and to analyse the significant interaction and dependency within the factors and for the comparison between samples. Statistical Software SPSS (ver. 25, IBM) was used. Results were displayed as line chart diagrams.
